# Long-Term Strength Development and Microstructural Characteristics of High-Content Cemented Soil Under Seawater Exposure

**DOI:** 10.3390/ma19071477

**Published:** 2026-04-07

**Authors:** Haoqiang Pan, Wenjun Wang, Jie Zhou, Xiao Cheng, Guangyang Hu

**Affiliations:** 1 MOE Key Laboratory of Soft Soils and Geoenvironmental Engineering, Institute of Geotechnical Engineering, Zhejiang University, Hangzhou 310058, China; 22312240@zju.edu.cn; 2 School of Civil Engineering & Architecture, NingboTech University, Ningbo 315100, China; chengx@nbt.edu.cn; 3 ZCONE High-tech Pile Industry Holdings Co., Ltd., Ningbo 315100, China; zhoujie@zcone.com.cn (J.Z.); huguangyang@zcone.com.cn (G.H.)

**Keywords:** high-content cemented soil, seawater exposure, long-term strength, microstructural evolution, micro–macro inconsistency

## Abstract

High-content cemented soils are critical for modern geotechnical technologies (e.g., pre-bored precast piles), yet their long-term durability remains underexplored. This study investigates the 28- to 365-day mechanical and microstructural evolution of high-content cemented silty clay under freshwater and seawater curing via UCS, SEM, MIP, and XRD. Under freshwater, cement content directly dictated strength, with the 8:2 mix reaching 24.31 MPa at 365 days. However, marginal efficiency analysis confirmed diminishing returns for excessive binder, establishing the 7:3 ratio as the optimal baseline. Seawater exposure induced a biphasic response: a 4.6% early strength gain at 28 days, followed by severe degradation (a 23.5% drop at 365 days). Concurrently, the failure mode shifted to macroscopic “pseudo-ductility,” with peak strain increasing from 2.37% to 3.04%. Crucially, a micro–macro inconsistency emerged: although seawater physically refined the pore structure (micropore proportion doubled to 30.2% at 90 days) via expansive salts filling mesopores, macroscopic strength declined. XRD confirmed this degradation coincides with severe long-term alkaline buffer (Ca(OH)_2_) depletion. Consequently, lifecycle durability assessments for high-binder marine systems must not rely solely on physical metrics like porosity, but adopt a coupled multi-factor framework prioritizing chemical stability.

## 1. Introduction

Rapid coastal infrastructural development has driven the adoption of advanced deep foundation systems [1], such as pre-bored precast concrete piles [2], the Metro Jet System (MJS) [3,4], and the Rodin Jet Pile (RJP) [4,5]. In conventional soil stabilization, cement content typically ranges from 10% to 30% by mass of dry soil [6,7,8]. However, these modern applications require highly flowable cement–soil slurries with mass-based cement contents (*a*_w_) often exceeding 40% to 80% to ensure ultra-high bearing capacity [9,10,11,12]. To explicitly distinguish these structurally over-saturated matrices from traditional stabilized soils, this study objectively defines them as “high-content cemented soils.” In these innovative systems, the enlarged high-content base provides the primary load-bearing capacity (up to 64–67%), making their structural integrity critical for engineering safety [13,14,15,16].

Extensive research on conventional soil–cement has established a reliable “porosity-strength synergy,” where hydration products fill initial voids, and reduced porosity directly translates to higher macroscopic strength [17,18,19,20]. However, the long-term durability of cementitious materials in marine environments is governed by complex chemo-mechanical coupling effects [21,22,23]. Recent studies on marine concrete and stabilized soils highlight that aggressive ions (e.g., chloride (Cl^−^), sulfate (${\mathrm{SO}}_{4}^{2-}$), and magnesium (Mg^2+^) ions) [24,25,26,27,28] induce severe degradation through sulfate-induced expansive cracking (e.g., ettringite and Friedel’s salt formation) [29,30] and magnesium-driven decalcification of the C-S-H gel [31,32,33]. Furthermore, the latest investigations explicitly confirm these severe deterioration mechanisms in both conventional marine soils and rigid concrete [34,35,36]. While the degradation trajectories of conventional soil–cement (characterized by rapid, simultaneous dissolution) and rigid marine concrete (characterized by delayed explosive cracking) are well-documented [34,37,38,39,40], the coupled chemo-mechanical response of high-content cemented soils remains theoretically ambiguous.

Despite the widespread application of high-content cemented soils in deep foundations, their long-term durability design remains largely empirical. Current engineering paradigms often extrapolate the classic “porosity-strength synergy”—established for conventional systems—to these cement-rich matrices. This implicitly assumes that a denser initial microstructure and a massive alkaline reserve inherently guarantee superior long-term erosion resistance. However, this direct extrapolation is fundamentally problematic. Existing research predominantly focuses on the macroscopic strength deterioration of conventional soil–cement or the expansive cracking of rigid marine concrete, leaving a critical knowledge gap regarding the complex chemo-mechanical coupling in high-content systems. Specifically, it remains largely unknown how the extreme initial compactness and robust alkaline buffer interact with the continuous ingress of marine ions over prolonged exposure (e.g., up to 365 days). Consequently, the long-term deterioration kinetics of high-content cemented soils remain theoretically ambiguous, making it unclear whether early-stage physical pore-filling by erosion products temporarily conceals the underlying chemical degradation of the cementing phases.

To address this knowledge gap and critically evaluate the limitations of relying solely on increasing cement content, this study systematically investigates the long-term (up to 365 days) chemo-mechanical deterioration of high-content cemented soil under seawater exposure. The primary scientific novelty lies in unraveling the competing effects of physical pore evolution and the underlying chemical degradation of the cementing phases in a high-content cemented soil system. The specific objectives are to: (1) quantitatively evaluate the mechanical evolution and establish an optimal cement efficiency threshold using macroscopic indices (e.g., the deformation modulus *E*_50_ and marginal efficiency *η*_c_); (2) elucidate the distinct phase-transformation-driven failure modes, specifically the anomalous increase in deformability despite macroscopic strength loss; and (3) unveil the underlying mechanisms driving the inconsistency between microstructural densification and macroscopic strength degradation through semi-quantitative, multi-scale microstructural tracking (via MIP, XRD, and SEM). Ultimately, the technical rationale of these findings is to provide experimental evidence for updating lifecycle durability assessments, moving design frameworks for marine foundations beyond simple physical porosity metrics to incorporate comprehensive chemo-mechanical stability.

## 2. Experimental Materials and Methods

### 2.1. Experimental Materials

**Figure 1:**
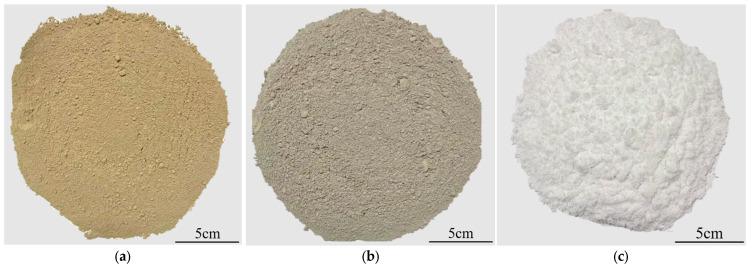
Test materials: (**a**) silty clay; (**b**) cement; (**c**) commercial sea salt.

Soil samples were obtained from the Phase III Project of the Hangyong Double Line in Beilun District, Ningbo, China at depths of 73.0–80.8 m. The material is a yellowish-green silty clay exhibiting a hard-plastic consistency, as shown in Figure 1. The basic physical properties of silty clay are presented in Table 1, respectively. P.O 42.5 Ordinary Portland Cement (OPC) purchased from Conch Company of China (Wuhu, China) was used as the binder; its major chemical constituents are detailed in Table 2.

Artificial seawater was prepared using commercial sea salt. To accurately simulate the aggressive marine exposure environment of Daxie Island, Ningbo, the preparation prioritized the precise control of primary reactive ions rather than relying solely on generic macroscopic indicators (such as bulk salinity). As established by extensive existing research, chloride (Cl^−^), sulfate (${\mathrm{SO}}_{4}^{2-}$), and magnesium (Mg^2+^) are identified as the key aggressive ions governing the deterioration pathways of cemented soil in marine environments. Therefore, the target concentrations of these specific ions (along with Ca^2+^ and Na^+^) were strictly calibrated based on local nearshore measurements. The detailed ionic composition is summarized in Table 3.

### 2.2. Experimental Program

To quantify the effects of cement content and curing age on the mechanical properties and microstructure of high-content cemented soil, five cement contents and six curing ages (28, 60, 90, 120, 180, and 365 days) were investigated. Curing periods exceeding 90 days were designated as the long-term curing stage.

Following the binder control protocols for pre-bored precast concrete piles, the mix proportion was defined as the volumetric ratio of cement slurry (*V*_C_) to the in situ soil volume (*V*_S_). The water–cement ratio of the slurry (*μ*) was fixed at 0.6. This volumetric ratio was converted to the conventional mass-based cement content (*a*_w_) using Equation (1):

(1) $$a_{w}=\frac{m_{c}}{m_{s}+m_{w}}$$

where *m*_c_ represents the mass of added cement; *m*_s_ denotes the mass of silty-clay powder; *m*_w_ indicates the total mass of mixing water, including both natural soil moisture and water in the cement slurry.

The mix proportion design in this study strictly refers to the Technical Specification for Pre-bored Precast Concrete Piles Foundation (DB33/T 1134-2017 [13]) and the actual construction parameters of the enlarged base in engineering practice. To meet the requirements of high fluidity and ultra-high bearing capacity at the bottom of pre-bored precast piles, five high-content volumetric ratios (*V*_C_:*V*_S_) ranging from 5:5 to 8:2 were established. Regarding the mixture selection strategy for the seawater erosion tests: considering the massive testing resources required for 365-day full-immersion curing and multi-scale microstructural tracking (MIP, SEM, XRD) across multiple ages, conducting an exhaustive parametric study in seawater for all five cement contents is impractical. Therefore, a step-by-step strategy was adopted. The macroscopic mechanical limits and marginal cement enhancement efficiency (*η*_c_) were first evaluated through the freshwater curing group, thereby establishing the 7:3 ratio as the “optimal engineering baseline” that balances mechanical performance and economic efficiency. Subsequently, the long-term seawater erosion and microstructural mechanism analyses were concentrated on this most representative optimal baseline. This approach aims to systematically explore and deduce the underlying degradation mechanisms of high-content cemented soil systems, rather than simply compiling a phenomenological parameter database.

The detailed testing program is outlined in Table 4.

### 2.3. Test Methods

#### 2.3.1. UCS Test

Unconfined compressive strength (UCS) tests were conducted using a universal testing machine in accordance with the Standard for Geotechnical Testing Method (GB/T 50123–2019 [41]). Cubic specimens (70.7 mm × 70.7 mm × 70.7 mm) were loaded at a displacement rate of 1.5 mm/min.

Three replicates were tested for each mix proportion. Data validity was determined based on the following criteria: (1) if the coefficient of variation (COV) was <20%, the mean value was reported; (2) if a single value deviated by >20%, it was discarded as an outlier, and the mean of the remaining two was used; and (3) if the variation exceeded 20% for all specimens, the test was repeated with fresh specimens. The final unconfined compressive strength (UCS) results are reported as the mean value ± standard deviation (SD). Accordingly, in all subsequent graphical representations of the mechanical test data, the data points correspond to the mean values, and the error bars denote the standard deviations.

#### 2.3.2. Microstructural Analysis

For microstructural characterization, samples were extracted from the core of intact specimens at each specified curing age. Rather than tracking localized surface erosion fronts, this core-sampling strategy was adopted to capture the homogeneous degradation of the bulk material. To rigorously ensure reproducibility and accurately capture the real-time microstructural state, it was imperative to arrest the hydration and erosion reactions immediately upon extraction. The core fragments were immediately immersed in absolute ethanol for 48 h to replace the pore water, followed by vacuum freeze-drying at −40 °C for 24 h.

The specific preparation and analytical procedures for each technique were as follows:

SEM: Small, representative fracture fragments (approximately 7 mm × 7 mm × 7 mm) were meticulously selected. Fresh, natural fracture surfaces were used instead of cut surfaces to preserve the true pore structure and spatial arrangement of the crystalline phases. The specimens were sputter-coated with gold for 60 s prior to imaging to enhance electrical conductivity. Micro-morphology was examined using a GeminiSEM 300 field emission scanning electron microscope, Carl Zeiss, Oberkochen, Germany. These qualitative visual observations were systematically cross-validated with the quantitative pore-size distribution data from MIP and the phase evolution data from XRD.

MIP: The dried fragments were carefully shaped into small blocks of approximately 1 cm^3^ to fit the penetrometer without inducing severe mechanical damage to the pore network. Pore-size distribution was determined using an AutoPore 9500 automated mercury porosimeter, Norcross, GA, USA. The maximum intrusion pressure was set to 60,000 psi, allowing for the detection of pore diameters covering the critical range of the cemented soil. To provide a rigorous quantitative evaluation, the continuous intrusion data were mathematically segmented into critical pore-size fractions: micropore (<0.01 μm), small pore (0.01–0.1 μm), mesopores (0.1–1 μm), and macropore (>1 μm).

XRD: The freeze-dried core samples were carefully prepared as intact bulk specimens to preserve their original structural characteristics and prevent mechanical damage to fragile crystalline phases. The samples were meticulously sectioned into small blocks with a maximum thickness of ≤5 mm and a diameter of ≤2 cm. The test surfaces were carefully smoothed to ensure they were strictly clean and flat, which is a critical requirement for accurate X-ray irradiation and to minimize scattering errors on bulk samples. Mineral phases and hydration products were identified using a Bruker D8 ADVANCE X-ray diffractometer, Bruker, Karlsruhe, Germany (scanned over a 2θ range of 5–90° at 2°/min). In addition to qualitative phase identification, a semi-quantitative comparative approach was adopted. Because all bulk specimens were scanned under strictly identical instrumental parameters and geometric surface conditions, the relative intensities (peak heights in counts) of characteristic diffraction peaks—such as Portlandite (Ca(OH)_2_) and Friedel’s salt—were directly compared to evaluate the chemical degradation trajectory.

### 2.4. Specimen Preparation

Specimen preparation and curing were conducted in accordance with the Specification for Mix Proportion Design of Cement Soil (JGJ/T 233–2011 [42]), as detailed below:

1. Air-dried soil was pulverized and sieved. The resulting powder was dry-mixed with cement for 1 min to ensure homogeneity.
2. The predetermined amount of water was added, followed by stirring at a low speed for 60 s and at a high speed for 30 s. To ensure uniformity, material adhering to the blades and mixer walls was scraped off between cycles; this mixing–scraping sequence was repeated three times.
3. The mixture was cast into 70.7 mm × 70.7 mm × 70.7 mm cubic molds in layers and vibrated for 3–5 min to eliminate entrapped air.
4. The specimen surfaces were leveled, and the molds were sealed with plastic film. Specimens were demolded after 48 h of curing at a constant temperature.
5. Subsequently, specimens were immersed in their respective curing environments (freshwater or seawater) at 20 ± 2 °C for duration of 28, 60, 90, 120, 180, and 365 days. For seawater curing, the solution was refreshed every 7 days during the first 28 days and every 30 days thereafter to maintain stable ion concentrations. It should be explicitly noted that this continuous full-immersion condition with periodic solution renewal constitutes an accelerated degradation environment.

**Figure 2:**
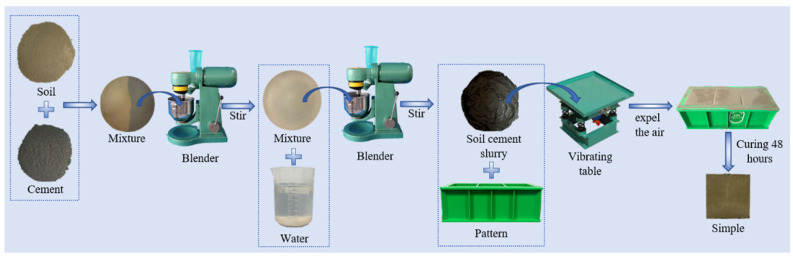
Specimen preparation flowchart.

The flowchart illustrating the specimen preparation process is presented in Figure 2.

## 3. Experimental Results

### 3.1. Effect of Cement Content and Curing Ages on UCS Under Freshwater Condition

#### 3.1.1. Stress–Strain Behavior

**Figure 3:**
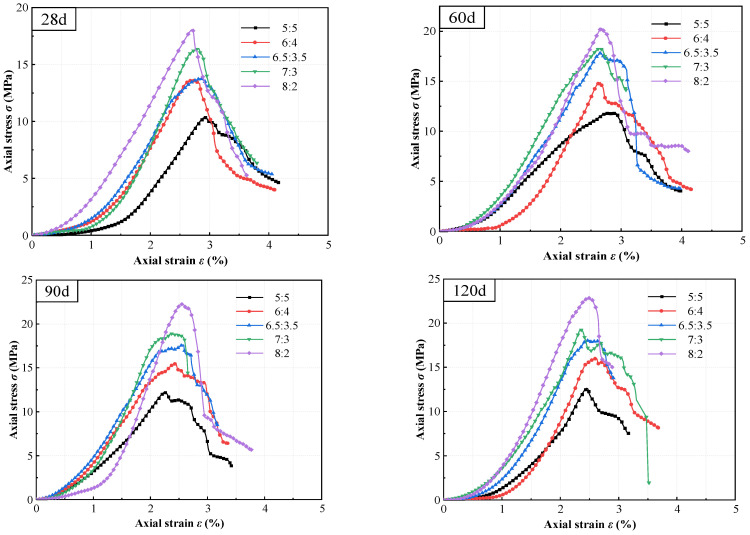

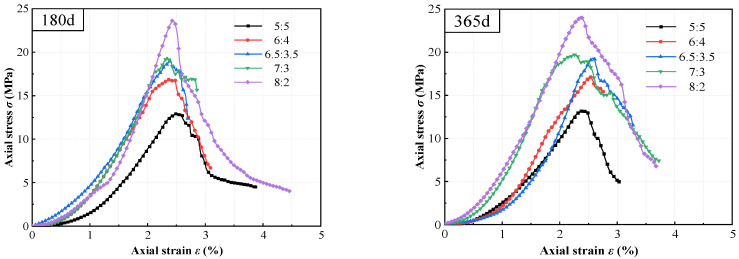
Stress–strain curves of high-content cemented soil under freshwater curing: effect of varying cement contents and progressive curing age.

To establish a reliable baseline for evaluating subsequent seawater degradation, the stress–strain behavior of high-content cemented soil under standard freshwater curing was first characterized. As illustrated in Figure 3, which details the responses under varying cement contents and progressive curing ages, the curves exhibited conventional compaction, linear hardening, and post-peak softening stages. Notably, increasing either the cement content (e.g., from *V*_C_:*V*_S_ = 5:5 to 8:2) or the curing age consistently enhanced the initial stiffness while reducing the peak strain (*ε_p_*). For instance, with cement content increased to *V*_C_:*V*_S_ = 8:2 and curing age extended to 365 days, the linear-hardening slope (initial stiffness) increased, whereas *ε_p_* decreased from 2.70% to 2.37%. This macroscopic response signifies a fundamental transition from a low-strength, ductile response to a high-strength, brittle failure mechanism under standard conditions.

Theoretically, this macroscopic embrittlement is governed by the volumetric over-saturation of hydration products. As supported by conventional soil–cement cementation theories [19,43,44], massive C-(A)-S-H gels rigidly interlock the soil aggregates and fill the initial voids, which significantly enhances the macroscopic tangent modulus but strictly restricts inter-particle sliding, thereby limiting post-yield deformability.

**Figure 4:**
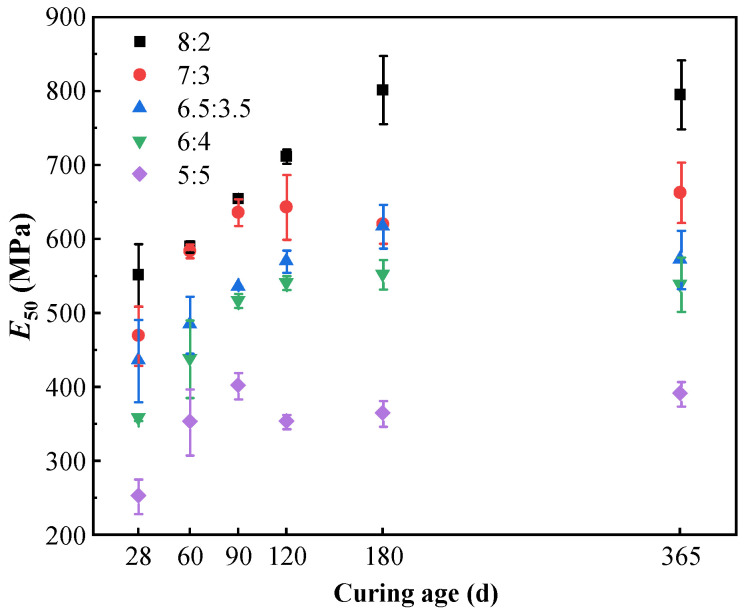
Evolution of deformation modulus (*E*_50_) of high-content cemented soil with different cement contents under freshwater curing.

To quantitatively evaluate this embrittlement behavior, the deformation modulus (*E*_50_), defined as the secant modulus at 50% of the peak unconfined compressive strength, was systematically extracted. As illustrated in Figure 4, *E*_50_ exhibits a consistent upward trend with both curing age and cement content. For instance, at the early age of 28 days, increasing the binder ratio from 5:5 to 8:2 resulted in a substantial surge in *E*_50_ from 252.9 MPa to 551.5 MPa. This rigidification becomes even more pronounced at long-term ages (365 days), where the *E*_50_ of the 8:2 group reaches nearly 800 MPa, completely doubling that of the 5:5 baseline (391.3 MPa). In the mechanics of cemented soils, such a dramatic multi-fold surge in stiffness (*E*_50_), coupled with the previously noted strict reduction in peak failure strain, serves as a classic quantitative indicator of material embrittlement. This explicitly confirms that higher cement contents drive the macroscopic failure mode from ductile to highly brittle.

#### 3.1.2. Peak Strength Evolution

As detailed in the methodology section, the coefficient of variation (COV) for all valid strength measurements was strictly controlled below 20% (averaging approximately 4.33%), demonstrating the high reliability and consistency of the mechanical data.

**Figure 5:**
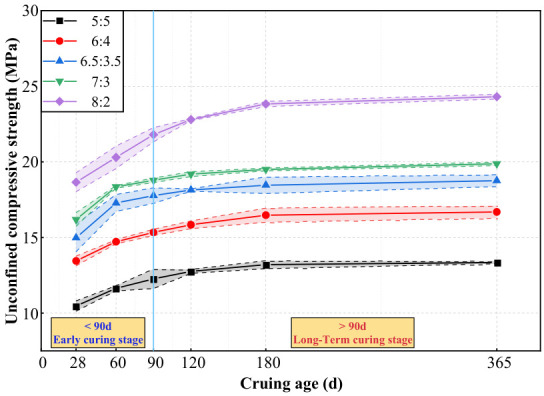
Variation curves of unconfined compressive strength of high-content cemented soil with curing age.

**Figure 6:**
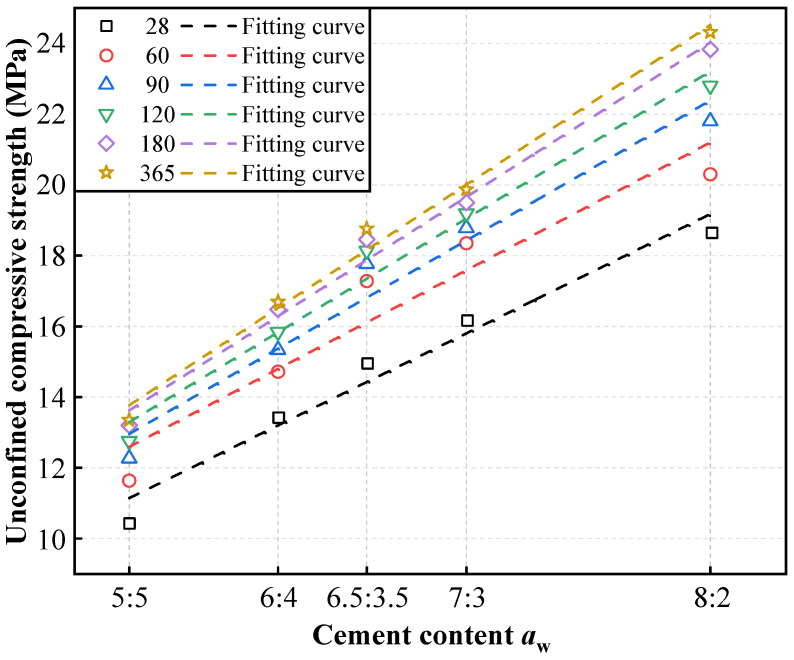
Variation curves of unconfined compressive strength of high-content cemented soil with cement content.

Figure 5 and Figure 6 present the unconfined compressive strength (UCS) evolution. Irrespective of the mix proportion, strength evolution exhibited a distinct biphasic pattern: a rapid early-stage gain (<90 days, driven by vigorous hydration) followed by a pronounced long-term deceleration (>90 days). By 365 days, the *V*_C_:*V*_S_ = 8:2 mixture reached 24.31 MPa, representing an 82.1% increase over the *V*_C_:*V*_S_ = 5:5 mix (13.35 MPa) and 45.7% increase over the *V*_C_:*V*_S_ = 6:4 mix (16.69 MPa).

To deeply evaluate the time-dependent hydration kinetics and the actual utilization rate of cement, two indices were introduced: the strength growth rate (*R*_T_) and the marginal cement enhancement efficiency (*η*_c_).

The strength growth rate (*R*_T_) is defined as the average rate of increase in unconfined compressive strength between two consecutive curing ages. *R*_T_ is calculated as follows:

(2) $$R_{T}=\frac{q_{u}(T_{i+1})-q_{u}(T_{i})}{T_{i+1}-T_{i}}$$

where *q*_u_(*T_i_*) and *q*_u_(*T_i_*_+1_) denote the unconfined compressive strengths at curing ages *T_i_* and *T_i_*_+1_ (MPa), respectively. The unit of *R*_T_ is MPa·d^−1^.

The marginal cement enhancement efficiency (*η*_c_) is defined as the strength increment per 1% increase in cement content relative to a baseline. *η*_c_ is calculated as follows:

(3) $$\eta_{c}=\frac{q_{u}(a_{i})-q_{u}(a_{0})}{a_{i}-a_{0}}$$

where *q*_u_(*a_i_*) and *q*_u_(*a*_0_) are the unconfined compressive strengths at cement contents *a_i_* and the baseline *a*_0_ (MPa), respectively. The unit of *η*_c_ is MPa·%^−1^.

**Figure 7:**
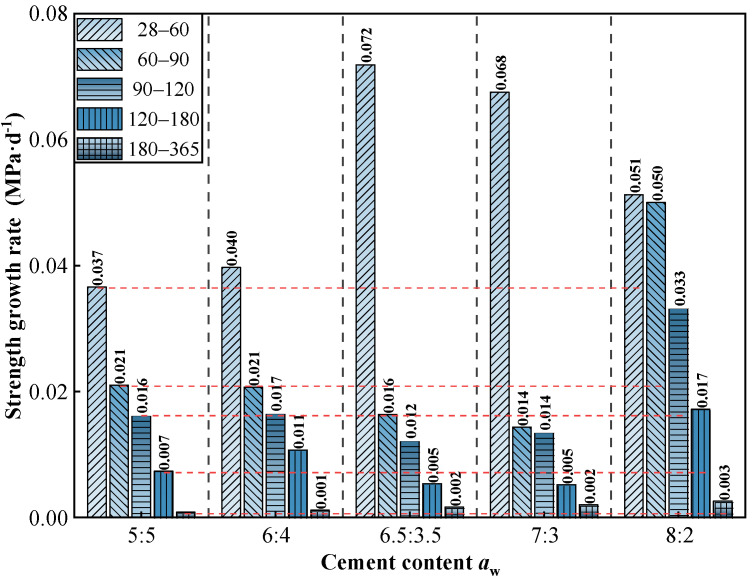
Strength growth rate of high-content cemented soil.

**Figure 8:**
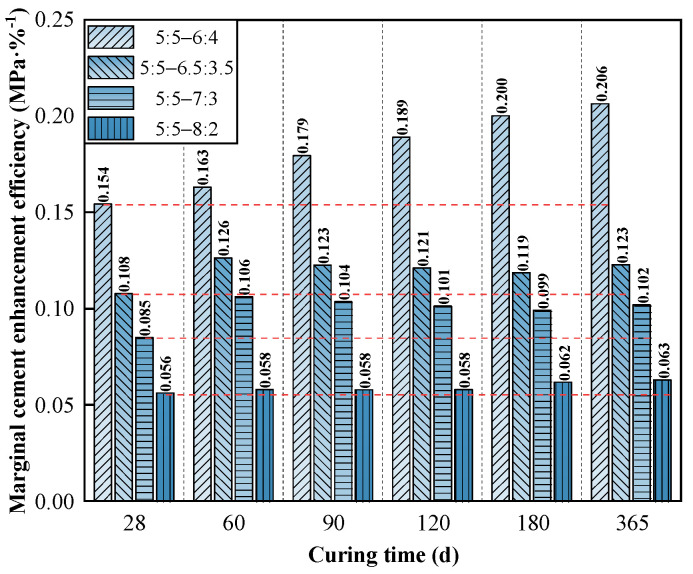
Marginal cement enhancement efficiency of high-content cemented soil.

As shown in Figure 7 and Figure 8, the strength development of all mixtures was heavily concentrated in the early curing stage (<90 days), with the strength growth rate (*R*_T_) diminishing rapidly and approaching zero after 180 days. Notably, an inverse relationship was observed between absolute strength and cement utilization efficiency. While the *V*_C_:*V*_S_ = 8:2 mixture achieved the highest absolute unconfined compressive strength, it yielded the lowest marginal enhancement efficiency (*η*_c_ = 0.06 MPa·%^−1^). Conversely, lower cement contents (e.g., *V*_C_:*V*_S_ = 6:4) demonstrated superior long-term efficiency (*η*_c_ = 0.206 MPa·%^−1^ at 365 days), indicating a more effective translation of additional binder into strength gains through hydration. These baseline indices confirm the diminishing marginal returns of excessive cement contents.

Physically, this diminishing marginal efficiency indicates that the high-content cement system approaches a hydration bottleneck. According to the structural formation theories of cemented soils [8,17], due to the restricted availability of free pore water and severe spatial confinement, excessive clinker fails to hydrate effectively, transitioning from an active cementing agent to an inert filler [38].

From a practical engineering perspective, while the baseline trend of strength increasing with cement content is widely acknowledged, the quantitative evolution of *η*_c_ provides a crucial optimization criterion for deep foundation design (e.g., pre-bored precast concrete piles). In conventional practice, engineers often attempt to blindly increase binder dosage to secure a higher safety margin against marine degradation. However, utilizing the *η*_c_ index explicitly warns against this approach. It can be practically applied to precisely determine the economic and mechanical optimal threshold (i.e., *V*_C_:*V*_S_ = 7:3). Exceeding this threshold not only wastes massive material costs and increases the carbon footprint, but also introduces excessive unhydrated clinker that could paradoxically jeopardize long-term structural durability in aggressive marine environment.

Jointly considering these mechanical tradeoffs alongside construction pumpability and cost efficiency, the *V*_C_:*V*_S_ = 7:3 mixture was definitively established as the optimal representative baseline for the subsequent seawater degradation analyses.

### 3.2. Effect of Seawater on UCS of High-Content Cemented Soil

#### 3.2.1. Effect on Strain–Stress Behavior: Emergence of Pseudo-Ductility

**Figure 9:**
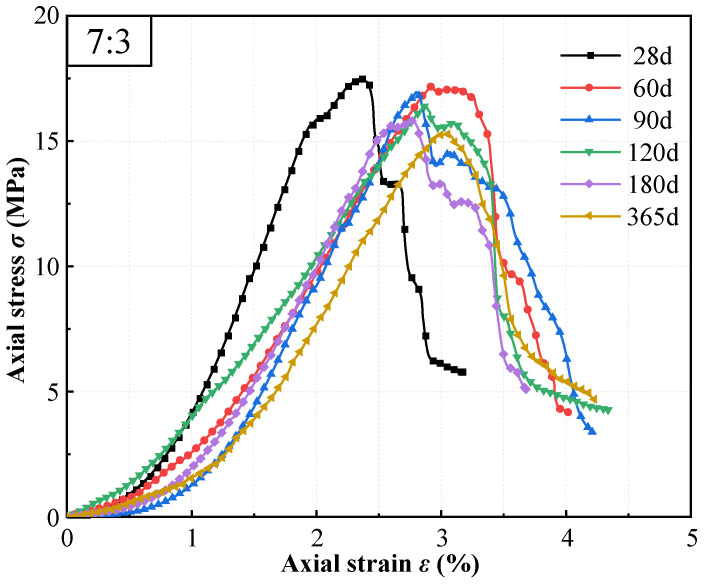
Stress–strain curves of high-content cemented soil with different curing ages in seawater.

Figure 9 illustrates the stress–strain behavior of high-content cemented soil (*V*c:*V*s = 7:3, *a*_w_ = 72.7%) cured in seawater. During the early curing stage (28 days), the curve morphology resembled that of freshwater-cured specimens, exhibiting high stiffness and brittle failure characteristics. In addition, the filling effect of salt crystals appeared to steepen the hardening segment. With extended seawater exposure, the curves displayed distinct post-peak behavior: stress decline became more gradual, and the descending branch became less steep, indicative of “pseudo-ductility.”

**Figure 10:**
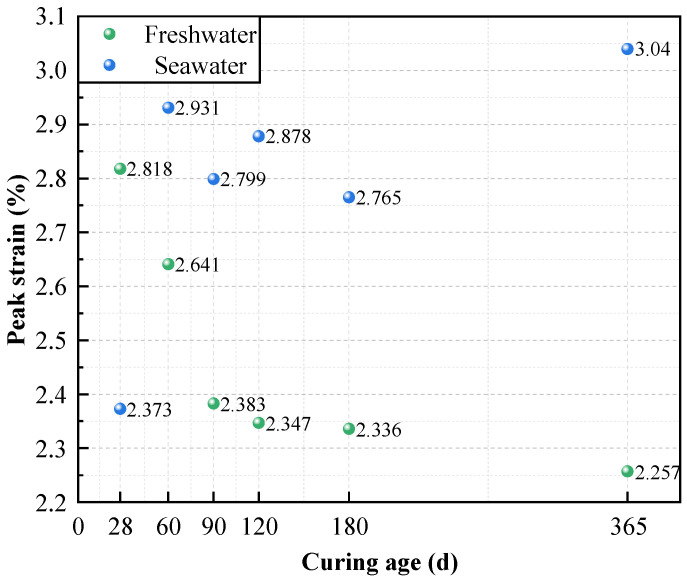
Peak strain of high-content cemented soil with different curing ages in seawater.

Figure 10 compares the peak strain variations in high-content cemented soil under different curing environments and ages. Results indicated that, unlike freshwater curing, the peak strain (*ε*_p_) of seawater-cured specimens increased with curing age, rising from 2.37% at 28 days to 3.04% at 365 days. This trend contrasted sharply with the monotonic decrease in *ε*_p_ observed under freshwater conditions. This phenomenon is attributed to the progressive deterioration of microstructural integrity due to seawater attack, resulting in stiffness degradation and enhanced deformability.

#### 3.2.2. Effect on Peak Strength: Dual-Stage Deterioration

**Figure 11:**
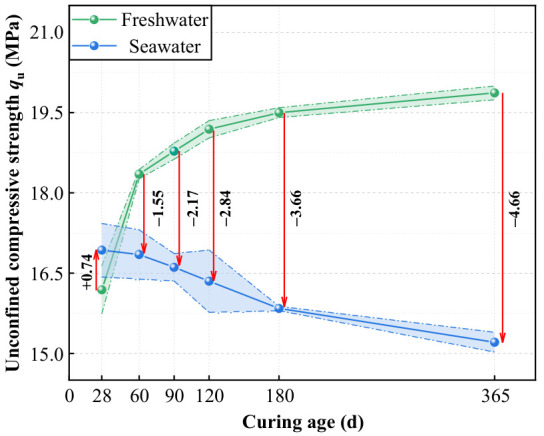
Variation curves of unconfined compressive strength of high-content cemented soil with curing age.

Figure 11 illustrates the variation in unconfined compressive strength (*q*_u_) of high-content cemented soil over curing age under both freshwater and seawater conditions. The results indicated that the impact of seawater exposure followed a distinct dual-stage pattern: early-stage enhancement followed by long-term deterioration.

To quantify the influence of seawater on the strength of the cemented soil, the relative strength change rate (*λ*) was introduced. Taking the strength under freshwater curing as the baseline, *λ* is defined as shown in Equation (4):

(4) $$\lambda =(q_{u}\;(S,\;x\;d)-q_{u}\;(F,\;x\;d))/(q_{u}\;(F,\;x\;d))(x=28,60,\cdots ,365)$$

where *q*_u_ (*F*, *x* d) and *q*_u_ (*S*, *x* d) represent the strengths of cemented soil cured in freshwater and seawater for *x* days, respectively. A positive value of *λ* indicates strength enhancement, whereas a negative value indicates strength degradation.

**Figure 12:**
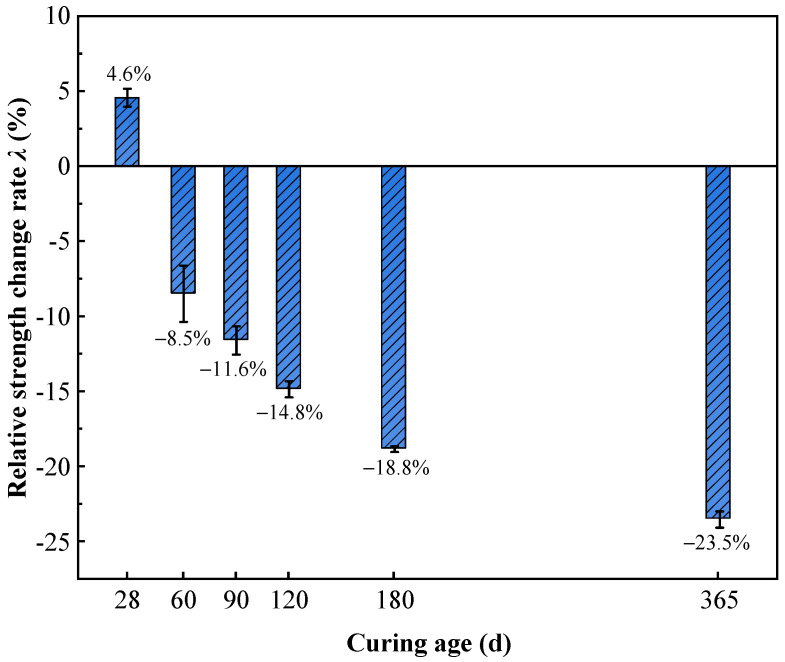
Relative strength change rate *λ* of high-content cemented soil at different curing ages.

Figure 12 presents the calculated relative strength change rates across different curing ages. At 28 days, seawater curing resulted in a 4.6% strength increase relative to the freshwater control. This early strength gain is attributed to the physical pore-filling effect of erosion products (e.g., Friedel’s salt and ettringite), as supported by microstructural observations [23,45]. However, with increasing curing age, *λ* shifted to negative values and exhibited a continuous decline. By 60 days, strength dropped 8.5% below the freshwater baseline, with the reduction reaching 23.5% by 365 days. These findings suggest that over time, chemical erosion-induced structural damage progressively outweighs the densification benefits of physical filling, ultimately becoming the dominant factor governing strength evolution [33,34,39].

### 3.3. Microstructural Evolution of High-Content Cemented Soil Under the Influence of Seawater

#### 3.3.1. Scanning Electron Microscope (SEM)

**Figure 13:**
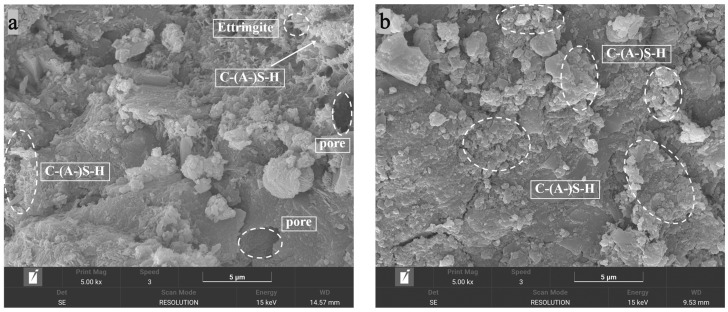

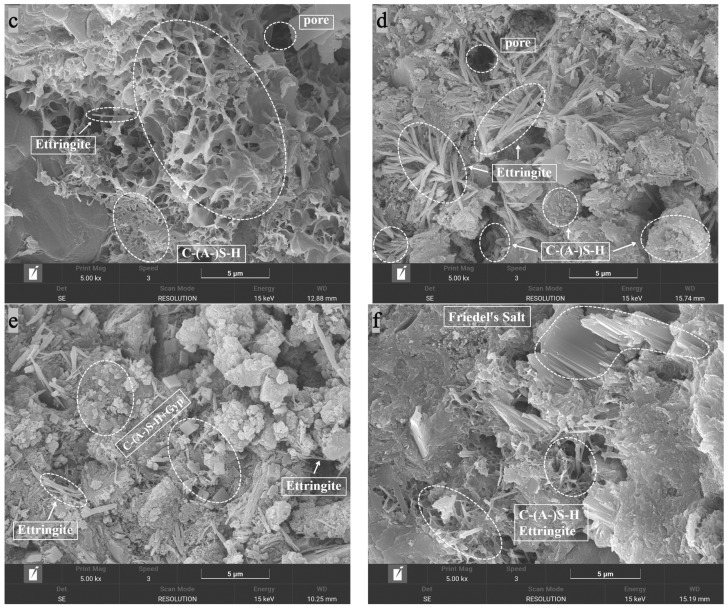
SEM micrographs of high-content cemented soil: (**a**) freshwater curing 28 d; (**b**) freshwater curing 90 d; (**c**) freshwater curing 365 d; (**d**) seawater curing 28 d; (**e**) seawater curing 90 d; (**f**) seawater curing 365 d.

Figure 13a–f present SEM micrographs of cemented soil specimens cured in freshwater and seawater, respectively, at curing ages of 28, 90, and 365 days (magnification: 5000×).

Extensive studies have shown that the strength development of cemented soil involves continuous processes such as cation exchange, flocculation, agglomeration, hydration, and pozzolanic reactions. The resulting hydration products—primarily C–S–H, C–A–H, C–A–S–H, and ettringite (AFt)—bind soil particles, thereby significantly enhancing mechanical strength [24,34]. While ettringite contributes to early strength development, its excessive formation can induce detrimental expansive stress [43].

At 28 days of freshwater curing [Figure 13a], the microstructure is relatively loose, featuring discrete hydration products morphologically consistent with needle-like AFt, and flocculent C–(A)–S–H gel. By 90 days [Figure 13b], the matrix densifies significantly as the C–(A)–S–H gel evolves into a continuous cementing matrix coating the soil aggregates, driving medium-term strength gain. At 365 days [Figure 13c], this gel matures into a robust, three-dimensional reticular network bridging the particles. This interconnected skeleton substantially enhances load-transfer efficiency, accounting for the continuous long-term strength development.

In the early stage of seawater curing [28 days, Figure 13d], the morphology resembles the freshwater baseline but features abundant radial AFt crystals within the pores. These ion-induced (Cl^−^, ${\mathrm{SO}}_{4}^{2-}$) expansive crystals provide an early physical filling effect, temporarily densifying the matrix and explaining the slightly higher macroscopic strength at 28 days. At 90 days [Figure 13e], fine granular gypsum emerges within the gel, indicating the ongoing physical filling by aggressive ions. By 365 days [Figure 13f], the microstructure undergoes a fundamental transformation marked by the proliferation of characteristic corrosion products. Based on morphological features and cross-validated with concurrent XRD patterns, the high-magnification field of view is predominantly occupied by massive, layered plate-like crystals inferred to be Friedel’s salt. These voluminous crystals intertwine with the reticular gel, densely occupying intergranular pores. Crucially, while this salt-crystal-dominated overfilling reduces porosity, it fundamentally lacks intrinsic cementing capacity and alters the intrinsic stiffness of the load-bearing skeleton. This micro-mechanism explains the observed macroscopic “pseudo-ductility” (high density combined with large post-yield deformation) and the pronounced strength degradation at later ages.

#### 3.3.2. Mercury Intrusion Porosimetry (MIP)

**Figure 14:**
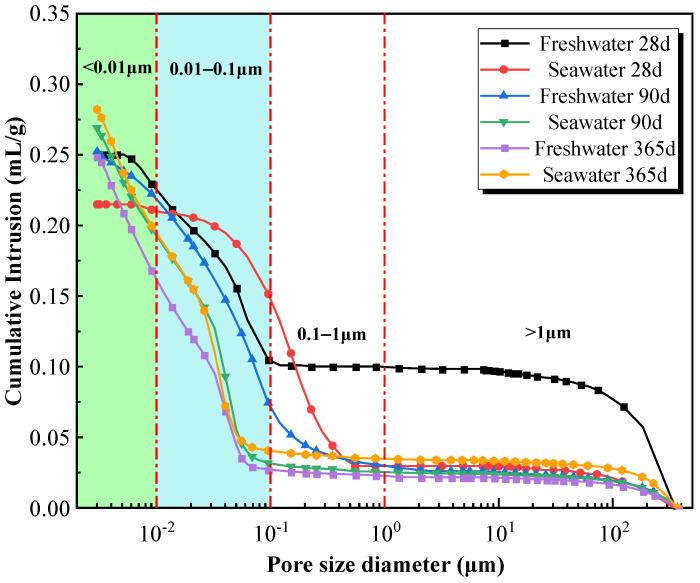
Cumulative mercury intrusion–pore size distribution curves of high-content cemented soil under freshwater/seawater curing.

**Figure 15:**
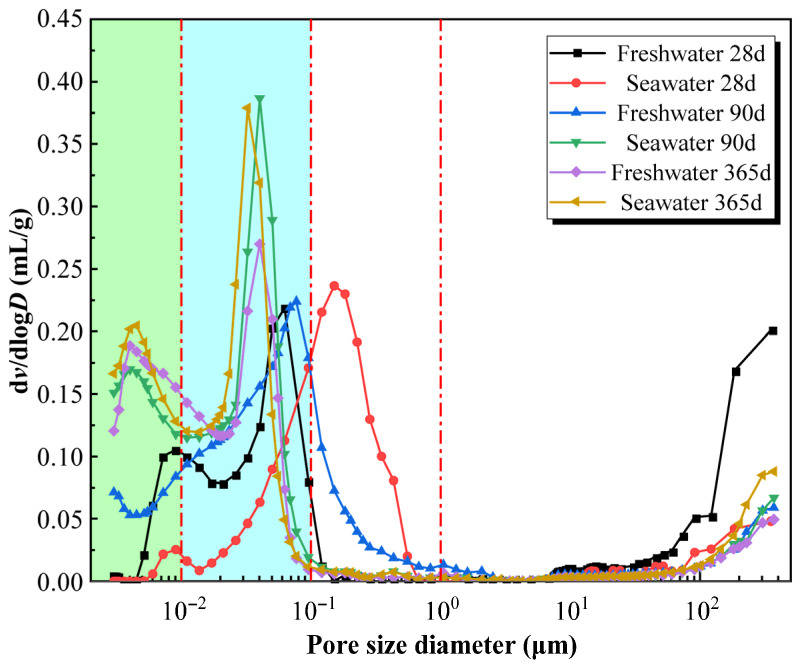
Pore size distribution curves of high-content cemented soil under freshwater/seawater curing.

**Figure 16:**
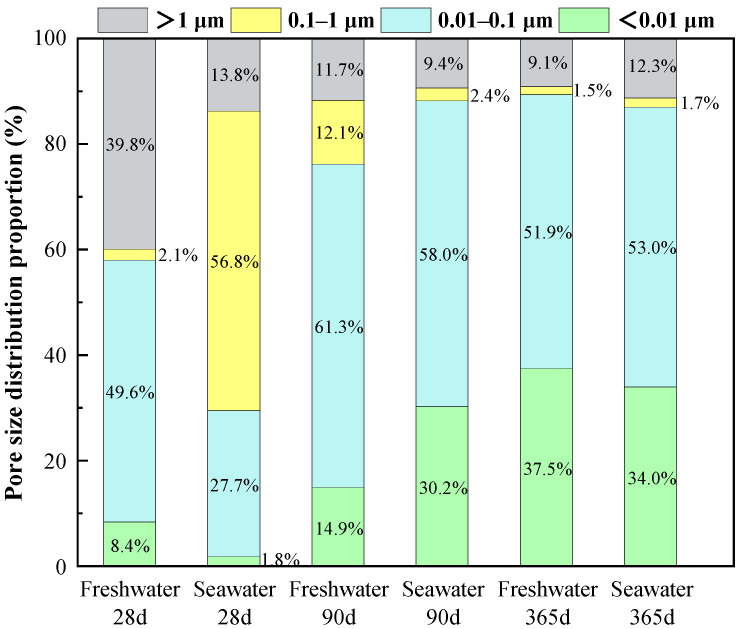
Histograms of pore size proportions of high-content cemented soil under freshwater/seawater curing.

Figure 14, Figure 15 and Figure 16 illustrate the pore structure evolution of high-content cemented soil under freshwater and seawater curing conditions. As shown in Figure 14, all cumulative mercury intrusion curves exhibited a characteristic inverted S-shaped profile. Figure 15 indicated that under freshwater curing, a continuous and significant densification was observed from 28 to 365 days. Initially, at 28 days, the microstructure was relatively loose, characterized by a high proportion of macropores (>1 μm) at 39.8% and only 8.4% micropores (<0.01 μm). As the curing age extended to 90 and further to 365 days, the primary steep-descent segment of the curve shifted significantly towards the micropore direction, accompanied by a reduction in total cumulative intrusion volume. As illustrated by the pore-size distribution (Figure 16), the proportion of micropores in the freshwater-cured specimen increased from 14.9% at 90 days to 37.5% at 365 days, whereas the mesopores proportion (0.1–1 μm) decreased from 12.1% to 1.5%. This significant pore refinement indicates that the continuous generation of C–(A)–S–H gel effectively fills larger voids, resulting in a dense cementing skeleton.

Conversely, seawater-cured specimens exhibited a distinct multiphase pore evolution: an immediate massive physical filling at 28 days, continued densification at 90 days, followed by subsequent structural deterioration at 365 days. In the initial 28-day stage, seawater exposure induced a dramatic pore-filling effect. Compared to the freshwater control (39.8% macropores), the macropore proportion in the 28-day seawater specimen sharply dropped to 13.8%, while the mesopore proportion (0.1–1 μm) spiked anomalously to 56.8%. This intense localized filling suggests that early-stage seawater-induced erosion products (e.g., ettringite and Friedel’s salt) rapidly precipitate and block larger defects, providing the microstructural basis for the macroscopic strength enhancement observed at 28 days. By 90 days, the primary steep-descent segment further shifted toward smaller diameters relative to the freshwater counterpart; the micropore proportion reached 30.2% (nearly double that of the freshwater group), while the mesopore proportion was reduced to 2.4%. Strikingly, as shown in Figure 16, the total cumulative volumes for the Seawater-365d and Freshwater-365d specimens are nearly identical. This demonstrates that seawater exposure does not necessarily increase overall porosity; rather, it fundamentally alters the nature of the solid skeleton.

Furthermore, the log differential intrusion curve (Figure 15) for the Seawater-365d specimen exhibits an exceptionally sharp and intense peak in the 0.01–0.1 μm range compared to the broader distribution of the freshwater counterpart. This distinct signature indicates a concentrated, uniform precipitation of crystalline salts rather than the formation of a continuous, multi-scale C–(A)–S–H gel network. Because the erosion products occupy pore volume but lack inherent cementing capacity and cannot compensate for the strength loss induced by skeletal degradation.

Although the micropore proportion further increased to 34.0% by 365 days, maintaining high overall density, the proportion of critical macropores (>1 μm) rebounded from 9.4% to 12.3%. More critically, the noticeable upturn at the extreme right of the d*v*/dlog*D* curve (>100 μm, Figure 15) provides direct evidence of microcrack formation. This coarsening reflects skeletal damage induced by long-term chemical attack, where expansive pressure from crystals and the dissolution of cementing phases promote microcrack propagation. These microstructural features provide direct evidence for the strength degradation and “pseudo-ductility” observed in macroscopic mechanical tests.

#### 3.3.3. X-Ray Diffraction (XRD)

**Figure 17:**
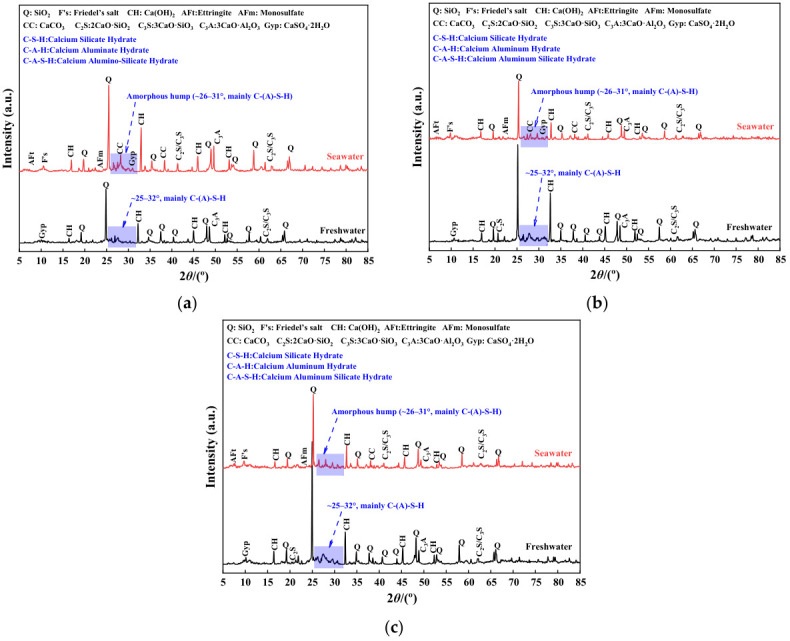
Comparison of XRD patterns for high-content cemented soil cured in freshwater and seawater at: (**a**) 28 d; (**b**) 90 d; (**c**) 365 d.

The evolution of phase composition was analyzed to elucidate the competitive interplay between hydration and ionic erosion under varying curing environments. Figure 17 presents the X-ray diffraction (XRD) patterns of high-content cemented soil cured in freshwater and seawater for 28, 90, and 365 days.

Under freshwater curing, the XRD patterns exhibit a characteristic trend of progressive hydration. From 28 to 90 days, diffraction peaks associated with unhydrated clinker phases (C_3_S/C_2_S) decreased markedly, while the intensity of the amorphous diffuse halo at 2θ ≈ 25–32° increased. This behavior indicates ongoing clinker dissolution accompanied by substantial formation of C–(A)–S–H gel and minor AFt, marking a stage of rapid strength development. By the long-term age of 365 days, the diffraction patterns stabilized; clinker peaks remained at low intensity while the diffuse halo remained prominent, indicating the system had entered a mature hydration stage. This evolution—from clinker consumption to gel formation and structural densification—provides the mineralogical basis for the steady long-term strength growth observed in freshwater-cured specimens.

In contrast, seawater fundamentally alters the phase-evolution pathway, driving a transition from hydration-dominated behavior to erosion-dominated processes. At 28 days, alongside conventional hydration products, distinct diffraction peaks corresponding to Friedel’s salt (e.g., at 2θ ≈ 11.2°), gypsum and AFt were detected. By 90 days, the intensities of these erosion-related peaks increased further, the absolute peak height of Friedel’s salt at 365 days exhibited a 23.6% relative increase compared to its 28-day level, confirming that active participation ${\mathrm{SO}}_{4}^{2-}$ and Cl^−^ in secondary reactions.

Unlike in freshwater specimens, the amorphous diffuse halo associated with C–(A)–S–H gel in seawater-cured samples did not intensify at 365 days, but instead exhibited slight attenuation. This suggests that under long-term saline exposure, C–(A)–S–H gel undergoes structural rearrangement, decalcification, or partial decomposition, which serves as a critical indicator strongly suggesting a high risk of decalcification.

Comparing phase assemblages between the two environments highlights the decisive role of ionic participation in governing hydration product composition. Portlandite (Ca(OH)_2_, or CH), acting as the alkaline reserve generated during hydration, serves as a key indicator of both hydration degree and chemical resistance. The results indicated that CH diffraction peak intensities (e.g., the primary peak at 2θ ≈ 18.1°) in seawater-cured specimens were markedly lower than those in their freshwater counterparts at identical ages.

Semi-quantitative tracking further reveals that between 90 and 365 days, freshwater-cured specimens exhibited a pattern of accumulation and stabilization, where CH was gradually consumed yet maintained at a stable residual level (recording approximately 1858 counts). Conversely, under seawater curing, the CH peak intensity experienced a drastic reduction, dropping from 2592 counts to merely 280 counts, indicating it was nearly depleted by 365 days. This semi-quantitatively observed long-term CH depletion eliminates the alkaline buffer. Consequently, this exhaustion acts as the fundamental chemical precursor that exposes the C–(A)–S–H gel to aggressive ionic attack, indirectly corroborating the aforementioned risk of gel decalcification and compromising the cementing matrix’s integrity. This micro-phase evolution—shifting from product coexistence at 90 days to matrix degradation at 365 days—corroborates the macroscopic observation that prolonged seawater immersion drives the transition from stability to progressive mechanical deterioration.

## 4. Discussion

### 4.1. Dominant Role of Cement Content in Governing the Theoretical Limit of Long-Term Strength

This study confirms that under freshwater curing, cement content governs the theoretical limit (or “ceiling”) of long-term strength, while curing age primarily dictates the rate at which this limit is approached. This was evidenced by the test results: at 365 days, increasing the cement content ratio from 5:5 to 8:2 resulted in a significant strength surge from 13.35 MPa to 24.31 MPa (an increase of 82.1%). In contrast, the temporal strength gains for a given mix proportion from 28 to 365 days ranged only between 22.73% and 30.55% (Figure 7 and Figure 8). This indicates that cement content establishes the strength potential, while curing time determines the extent to which this potential is realized. This finding quantitatively extends the classical theory that binder dosage controls strength potential [14,38].

Furthermore, the stress–strain evolution reveals the enhanced stiffness and intensified brittleness associated with high cement content. As cement content increases, the linear elastic segment steepens significantly, quantitatively evidenced by the substantial surge in the deformation modulus (*E*_50_) evaluated in Figure 4, while peak strain decreases noticeably (e.g., dropping from 2.704% to 2.367% in the 8:2 group; see Figure 3 and Figure 10). Based on the microstructural densification observed in our SEM images and established soil–cement theories [10], this macroscopic behavior is attributed to the high concentration of hydration products (C-(A)-S-H), which constructs a more continuous and rigid cementing skeleton. While this significantly improves compressive strength, it restricts inter-particle sliding, resulting in a failure mode characterized by typical “high strength–high brittleness”.

### 4.2. Time-Dependent Deterioration of Strength and “Pseudo-Ductility” Under Seawater Environment

Seawater-induced damage to the strength and stability of cemented soil exhibits a significant time-cumulative effect, distinct from freshwater conditions. Based on the analysis of strength data and the relative strength change rate (*λ*), the influence of seawater presents a distinct biphasic characteristic: “early filling enhancement” versus “late chemical deterioration.”

**Early Stage (28 d)**: The strength of the seawater group (16.93 MPa) slightly exceeded that of the freshwater group (*λ* = 4.6%). As directly supported by the pore size refinement in our MIP data and the observation of crystalline clusters in SEM, this is attributed to the physical filling effect of reaction products (e.g., Friedel’s salt), which temporarily compensates for pore defects.

**Long-term Stage (365 d)**: The strength showed significant regression, dropping substantially, with *λ* falling to −23.5%. This confirms that over time, skeletal damage induced by chemical erosion progressively outweighs the densification benefits of physical filling. Similar “early stability, late deterioration” phenomena have been widely reported in saline environments, linked to mechanisms of Mg^2+^ -induced Ca^2+^ leaching and C-S-H decalcification [34,39]. However, for high-content cemented soil, it is notable that despite strength attenuation occurred, no Magnesium Silicate Hydrate (M-S-H) was detected in XRD patterns. As semi-quantitatively supported by the XRD analysis in Section 3.3.3, where the Ca(OH)_2_ peak intensity dropped precipitously (from 2592 to 280 counts), this indicates that the extremely high initial cement content provides a massive alkaline reserve. This reserve acts as a sacrificial chemical barrier that potentially delays the complete transformation of C-S-H into non-cementitious M-S-H, even though the structural integrity of the active gel is already chemically compromised.

Meanwhile, seawater curing significantly altered the material’s failure mode. Unlike the freshwater group, where peak strain decreased with age, the peak strain of seawater group specimens anomalously increased from 2.37% (28 d) to 3.04% (365 d), accompanied by a gentler post-peak softening response (see Figure 9). This “pseudo-ductility” represents not a genuine improvement in toughness, but rather a manifestation of impaired structural integrity. It is mechanistically inferred that the high risk of partial C-S-H decalcification (triggered by the aforementioned severe depletion of the alkaline buffer), coupled with Mg^2+^-induced microcracking reduce the skeleton tangent modulus, resulting in greater deformability during failure.

### 4.3. The Paradox Between Pore Refinement and Strength Degradation

Microscopic results reveal a critical counter-intuitive phenomenon: pore size refinement does not necessarily translate into improved macroscopic strength. To quantitatively demonstrate this micro–macro inconsistency, a stage-by-stage comparative analysis is established. At 28 days, early physical filling drastically reduced macropores (>1 μm) by 65.3% (from 39.8% to 13.8%), providing the precise microstructural basis for the 4.6% strength enhancement. However, a quantitative paradox emerged at 90 days. MIP data show that at 90 days, the micropore proportion in seawater group specimens (30.2%) significantly exceeded that of the freshwater group (14.9%), indicating superior microscopic compactness prematurely (Figure 14, Figure 15 and Figure 16). However, the corresponding macroscopic strength was lower. This paradoxical “micro-macro inconsistency” demonstrates that in seawater environments, evaluating mechanical properties of high-content cemented soil solely based on porosity is fundamentally flawed. Furthermore, from 90 to 365 days, the proportion of critical macropores (>1 μm) rebounded from 9.4% to 12.3%, a 30.8% relative expansion that quantitatively correlates with the severe 23.5% macroscopic strength attenuation.

To decode the mineralogical basis for this inconsistency, XRD and SEM observations must be systematically integrated. To ensure a rigorous methodological interpretation, it is acknowledged that while SEM provides essential representative visual evidence, it is inherently qualitative. Furthermore, the absence of EDS and TG/DTG in this study limits direct elemental mapping and the absolute quantification of phase contents. To effectively mitigate these limitations, the following mechanistic inferences are derived by strictly cross-validating the representative SEM micro-morphologies with quantitative phase and pore data from XRD and MIP, alongside macroscopic mechanical responses. Guided by this cross-validation framework, the representative SEM micrographs [Figure 13d,e] reveal that pore spaces are densely packed with crystalline phases. While the exact elemental composition requires future EDS verification, these voluminous crystals—coupled with the distinct diffraction peaks identified in the concurrent XRD patterns (Figure 17)—are reasonably inferred to be Friedel’s salt and gypsum induced by Cl^−^ and ${\mathrm{SO}}_{4}^{2-}$. Although these crystals densely fill inter-granular pores (physical effect), they fundamentally lack the intrinsic cementing capacity of the C-(A)-S-H gel (chemical effect) and introduce interfacial heterogeneity.

**Figure 18:**
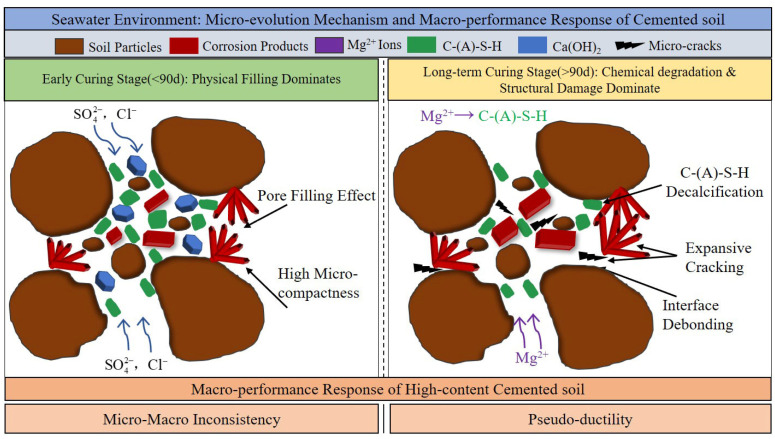
Schematic diagram of the long-term strength deterioration mechanism of high-content cemented soil under seawater environment.

Crucially, XRD patterns show that long-term seawater exposure (365 days) leads to the severe attenuation of the Ca(OH)_2_ (CH) diffraction peaks, as established in Figure 17, the CH peak intensity plummeted drastically from 2592 to merely 280 counts. While precise CH consumption rates await future TG/DTG quantification, this observed depletion indicates the exhaustion of the alkaline buffer. Concurrently, the stagnation of the C-(A)-S-H diffuse halo in XRD suggests that the gel matrix undergoes structural rearrangement. Consequently, it is deduced that the strength reduction in high-content cemented soil is not controlled by pore refinement, but is dominated by a synergistic deterioration mechanism: (1) the progressive chemical breakdown of the cementing matrix (a highly probable indirect inference drawn from the severe CH consumption, Mg^2+^ attack, and subsequent C-S-H decalcification risk); (2) structural heterogeneity and micro-cracking induced by expansive crystals; and (3) the late-stage rebound of defective macropores (>1 μm), which increased from 9.4% to 12.3% as captured by MIP. This shifts the load-bearing mechanism from “continuous gel cementation” to “defect-controlled failure,” ultimately manifesting as long-term strength attenuation and “pseudo-ductility”. The proposed mechanism is illustrated in Figure 18.

### 4.4. Comprehensive Mechanism and Engineering Implications

To clarify the specific degradation mechanisms observed in this study, a direct comparison with existing literature on conventional marine concrete and low-content cemented soil is necessary. In marine concrete exposed to saline environments [35,36], the phenomenon of “micro-macro inconsistency” is frequently reported; however, it is typically driven by the delayed formation of expansive products (e.g., delayed ettringite formation) within a highly rigid matrix. This leads to sudden macro-cracking despite an initially dense microstructure. Conversely, in traditional soil–cement systems (typically 10–30% cement content) [6,7,8,33,45], physical porosity coarsening and macroscopic strength loss occur almost simultaneously as the sparse cementing gel matrix simply dissolves under ionic attack.

In contrast, the high-content cemented soil in this study exhibits a distinctly different deterioration trajectory. Due to the extremely high initial binder content, the early-stage ingress of marine ions triggers intense localized crystallization that artificially refines the pore structure (quantitatively evidenced by the micropore proportion soaring to 30.2% at 90 days, nearly double that of the freshwater controls). This intense early-stage physical densification temporarily conceals the concurrent progression of underlying chemical damage—specifically, the severe alkaline depletion semi-quantitatively captured in the XRD analysis. Unlike marine concrete, the failure here is not characterized by explosive macro-cracking, but rather by the progressive “pseudo-ductile” softening of the load-bearing skeleton. This direct, quantitative comparison explicitly distinguishes the deterioration kinetics of high-content cemented soil from both conventional concrete and traditional soil stabilization, relying strictly on observed microstructural and chemical evidence rather than generalized models.

However, this high content does not fundamentally arrest the dominant chain of long-term strength deterioration in aggressive marine environments (i.e., ionic penetration→alkaline depletion→cementing phase decalcification→microcrack propagation→macroscopic strength attenuation). This finding has critical implications for coastal engineering: when evaluating the long-term durability of high-content cemented soil (e.g., pre-bored precast concrete piles), reliance must not be placed solely on physical indicators such as porosity. Instead, lifecycle assessments must prioritize the chemical stability of the cementing phases and the integrity of microscopic load-bearing paths. When extrapolating these findings to engineering practice, the distinction between laboratory accelerated exposure and in situ service conditions must be fully acknowledged. The continuous immersion and forced solution renewal in this study rapidly replenished aggressive ions, artificially accelerating the deterioration cascade (e.g., alkaline depletion and decalcification). In actual coastal engineering, the degradation rate is typically much slower due to complex boundary conditions, varying saturation degrees, and limited mass transport. Therefore, the primary contribution of this study lies in decoding the overarching mechanistic trajectory and the micro–macro failure modes of high-binder systems, rather than providing a precise quantitative prediction of their field lifespan.

## 5. Conclusions

This study systematically investigated the long-term strength (up to 365 days) and microstructural evolution of high-content cemented soil under seawater exposure. Based strictly on the experimental results (via UCS, SEM, MIP, and XRD), the following conclusions are drawn:

(1) **Strength, Deformation, and Efficiency in Freshwater**: Cement content directly dictates the theoretical “ceiling” of macroscopic strength, with the 8:2 mix reaching 24.31 MPa at 365 days (an 82.1% increase over the 5:5 mix). However, higher contents consistently decreased peak strain, inducing a distinctly brittle failure mode, quantitatively evidenced by a substantial surge in the deformation modulus (*E*_50_). Furthermore, analysis of the strength growth rate (*R*_T_) and marginal cement enhancement efficiency (*η*_c_) confirmed diminishing returns for excessive binder: while the 8:2 mixture achieved the highest strength, it yielded the lowest marginal efficiency (*η*_c_ = 0.06 MPa·%^−1^). Consequently, the 7:3 ratio was established as the optimal baseline, effectively balancing mechanical performance and economic efficiency.
(2) **Biphasic Seawater Response and Pseudo-ductility:** Seawater exposure induces a distinct dual-stage strength evolution: early enhancement followed by long-term deterioration. At 28 days, physical pore-filling resulted in a slight strength gain (*λ* = 4.6%). However, by 365 days, significant degradation occurred (*λ* = −23.5%). Concurrently, the failure mode shifted from brittle fracture to “pseudo-ductility”—characterized by an anomalous increase in peak strain (from 2.37% to 3.04%) and a gentler post-peak softening response. This shift reflects a progressive loss of structural integrity rather than a genuine improvement in material toughness.
(3) **Pore Structure–Strength Paradox (Micro–Macro Inconsistency):** A critical inconsistency between microstructural densification and macroscopic strength was identified under long-term seawater conditions. Despite significant early pore refinement at 90 days (micropore proportion reaching 30.2%), macroscopic strength paradoxically regressed. This intense early-stage physical densification temporarily conceals the concurrent progression of chemical damage. Semi-quantitative XRD explicitly confirmed this underlying degradation via a massive depletion of the Ca(OH)_2_ alkaline buffer (plummeting from 2592 to 280 counts), triggering a high risk of subsequent decalcification.
(4) **Engineering Implications:** The observed micro–macro inconsistency demonstrates that physical pore refinement (induced by expansive salts filling the mesopores) does not prevent macroscopic strength loss under long-term marine exposure. Consequently, for high-binder marine foundations such as pre-bored precast concrete piles, lifecycle durability assessments should not rely solely on physical metrics (e.g., porosity). Instead, design standards must adopt a comprehensive multi-factor evaluation framework, coupling macroscopic physical compactness with the long-term chemical stability of the cementing phases to ensure reliable lifecycle safety predictions.

## 6. Limitation and Future Work

The findings of this study are based on specific experimental boundaries. The mechanical data and microstructural characteristics were obtained from a single representative optimal mixture (*V*_C_:*V*_S_ = 7:3) subjected to an accelerated, artificial full-immersion seawater environment. Additionally, phase identifications and the depletion of the alkaline buffer were primarily derived from qualitative XRD and SEM/MIP observations. Future research must incorporate a broader range of high-binder contents to develop robust analytical degradation models, evaluate long-term in situ field exposure, and utilize advanced quantitative characterization techniques (such as EDS and TG/DTG) to rigorously quantify specific elemental and phase alterations (e.g., specific C-S-H variations).

## Figures and Tables

**Table 1:** Basic physical properties of silty clay.

Water Content (%)	Wet Density (kN·m^−3^)	Particle Specific Gravity	Void Ratio	Plastic Limit (%)	Liquid Limit (%)
26.14	1.97	2.72	0.738	24.8	42.8

**Table 2:** Major chemical constituents of cement.

Composition	SiO_2_	CaO	Al_2_O_3_	Fe_2_O_3_	K_2_O	Na_2_O	SO_3_	MgO	Ignition Loss (%)
Content (%)	25.0	51.42	8.26	4.03	2.17	0.79	2.51	3.71	1.16

**Table 3:** The content of the major ions in artificial seawater.

Major Ions (mg/L)	Artificial Seawater	Daxie Island’s Seawater
Ca^2+^	486.0	481.0
Mg^2+^	872.2	846.1
Na^+^	8140.0	7720.0
Cl^−^	15,422.1	14,713.0
${\mathrm{SO}}_{4}^{2-}$	1625.0	1450.0
${\mathrm{HCO}}_{3}^{-}$	24.4	36.6

**Table 4:** Test program.

Cement Content		*m*_c_:*m*_w_:*m*_s_	Curing Environment	Curing Age/d	Test Type
*V*c:*V*s	*a*_w_/%				
8:2	93.1	2.7:1.8:1.0	Freshwater	28, 60, 90, 120, 180, 365	UCS
7:3	72.7	1.6:1.2:1.0			
6.5:3.5	61.7	1.2:1.0:1.0			
6:4	53.6	1.0:0.8:1.0			
5:5	40.0	0.7:0.6:1.0			
7:3	72.7	1.6:1.2:1.0	Seawater	28, 60, 90, 120, 180, 365	UCS
			Freshwater Seawater	28, 90, 365	SEM, XRD MIP

## Data Availability

The original contributions presented in this study are included in the article. Further inquiries can be directed to the corresponding author.
